# Imaging angiogenesis in patients with head and neck squamous cell carcinomas by [^68^Ga]Ga-DOTA-E-[c(RGDfK)]_2_ PET/CT

**DOI:** 10.1007/s00259-020-04766-2

**Published:** 2020-03-20

**Authors:** D. Lobeek, M. Rijpkema, S. Y. A. Terry, J. D. M. Molkenboer-Kuenen, L. Joosten, E. A. J. van Genugten, A. C. H. van Engen-van Grunsven, J. H. A. M. Kaanders, S. A. H. Pegge, O. C. Boerman, W. L. J. Weijs, M. A. W. Merkx, C. M. L. van Herpen, R. P. Takes, E. H. J. G. Aarntzen, W. J. G. Oyen

**Affiliations:** 1 Department of Radiology and Nuclear Medicine, Radboud University Medical Center, P.O. Box 9101, 6500 HB Nijmegen, The Netherlands; 2 Department of Imaging Chemistry and Biology, King’s College London, London, UK; 3 MIRA Institute for Biomedical Technology and Technical Medicine, University of Twente, Enschede, The Netherlands; 4 Department of Pathology, Radboud University Medical Center Nijmegen, Nijmegen, The Netherlands; 5 Department of Radiotherapy, Radboud University Medical Center Nijmegen, Nijmegen, The Netherlands; 6 Department of Oral and Maxillofacial Surgery, Radboud University Medical Center, Nijmegen, The Netherlands; 7 Department of Medical Oncology, Radboud University Medical Center Nijmegen, Nijmegen, The Netherlands; 8 Department of Otolaryngology/Head and Neck Surgery, Radboud University Medical Center, Nijmegen, The Netherlands; 9 Department of Biomedical Sciences, Humanitas University, Milan, Italy; 10 Department of Radiology and Nuclear Medicine, Rijnstate Hospital, Arnhem, The Netherlands

**Keywords:** RGD-PET/CT, Angiogenesis, Squamous cell carcinoma, Head and neck cancer, α_v_β_3_ integrin

## Abstract

**Purpose:**

Angiogenesis plays an important role in the growth and metastatic spread of solid tumours and is characterised by the expression of integrins on the cell surface of endothelial cells. Radiolabelled RGD peptides specifically target angiogenesis-related α_v_β_3_ integrins, expressed on the activated endothelial cells of sprouting blood vessels. Here, we validated the feasibility of ^68^Ga[Ga]-DOTA-E-[c(RGDfK)]_2_ (^68^Ga-RGD) PET/CT to visualise angiogenesis in patients with oral squamous cell carcinoma (OSCC).

**Methods:**

Ten patients with OSCC and scheduled for surgical resection including elective neck dissection received an intravenously administration of ^68^Ga-RGD (42 ± 8 μg; 214 ± 9 MBq). All patients subsequently underwent dynamic (*n* = 5) or static PET/CT imaging (*n* = 5) for 60 min or for 4 min/bed position at 30, 60 and 90 min after injection, respectively. Quantitative tracer uptake in tumour lesions was expressed as standardised uptake values (SUV). Additionally, tumour tissue was immunohistochemically stained for α_v_β_3_ integrin to assess the expression pattern.

**Results:**

^68^Ga-RGD tumour accumulation was observed in all patients. At 60 min post injection, tumour SUV_max_ ranged between 4.0 and 12.7. Tracer accumulation in tumour tissue plateaued at 10 min after injection. Uptake in background tissue did not change over time, resulting in tumour-to-muscle tissue of 6.4 ± 0.7 at 60 min post injection.

**Conclusions:**

^68^Ga-RGD PET/CT of α_v_β_3_ integrin expression in OSCC patients is feasible with adequate tumour-to-background ratios. It will provide more insight in angiogenesis as a hallmark of the head and neck squamous cell carcinomas’ tumour microenvironment.

**Trial registration:**

https://eudract.ema.europa.eu no. 2015-000917-31

**Electronic supplementary material:**

The online version of this article (10.1007/s00259-020-04766-2) contains supplementary material, which is available to authorized users.

## Introduction

Head and neck squamous cell carcinomas of the mucosal surfaces (HNSCC) are the most common type of all head and neck cancers, with approximately 890,000 new cases in 2018 worldwide [1]. Current treatment options for HNSCC depend on the primary site of the cancer, stage, resectability, and wish to preserve functional outcome [2, 3]. In oral squamous cell carcinomas (OSCC), surgery with on indication followed by (chemo)radiotherapy is the first choice of treatment, whereas in most other sites in the head and neck region, (chemo)radiation is the first choice. Furthermore, for locally advanced and recurrent diseases, several advances to treat them with molecular targeted therapies have been made [4]. Despite these advances, treatment is often not effective in many patients, while still causing significant toxicity. This illustrates the importance of identifying predictive biomarkers to stratify patients who are most likely to benefit from (molecular) targeted therapies. A better understanding of the tumour microenvironment and its effect on treatment resistance could further improve the management of HNSCC.

Angiogenesis, the formation of new blood vessels, is a hallmark of tumour progression and metastatic spread [5, 6]. Its role in HNSCC has been studied intensively, and various strategies inhibiting the cross talk between signalling pathways involved in the growth and metastasis of HNSCC have been revealed as potential therapeutic target [7, 8].

Integrins play a key factor in controlling cell-cell signalling during angiogenesis and may serve as an angiogenesis-related biomarker [5]. The most extensively studied integrin, α_v_β_3_, is expressed on activated endothelial cells during normal tissue regeneration and becomes aberrantly expressed in cancers, subsequently leading to tumour progression and metastatic spread [9]. Whereas α_v_β_3_ integrins can be expressed on both tumour cells and tumour-associated neovasculature, it is a selective target of neovasculature in HNSCC [10]. Thus, it may provide important information on tumour microvasculature in HNSCC. Using positron emission tomography/computed tomography (PET/CT), radiolabelled peptides targeting the α_v_β_3_ integrins enable the noninvasive and early evaluation of microenvironmental changes associated with treatment success or failure and may provide a better understanding of angiogenesis-related tumour characteristics.

Integrin α_v_β_3_ can be targeted by radiotracers containing the arginine-glycine-aspartic acid (RGD) amino acid sequence present in extracellular matrix proteins such as vitronectin [11]. In recent years, many studies focused on the potential of molecular imaging with RGD-containing peptides targeting α_v_β_3_ integrins [12]. Several monomeric and multimeric ^68^Ga-labelled RGD compounds have been developed and were studied in glioblastoma [13], cervical and ovarian cancer [14] and lung adenocarcinoma [15]. The dimeric radiotracer [^68^Ga]Ga-DOTA-E-[c(RGDfK)]_2_ (^68^Ga-RGD) has preclinically shown its potential for imaging of angiogenesis in HNSCC [16, 17], but only limited data from clinical studies is available. Therefore, we aim to study the safety, biodistribution and kinetics of ^68^Ga-RGD and characterise α_v_β_3_ integrin expression patterns in patients prior to primary surgical resection of an OSCC.

## Patients and methods

### Patients

This prospective study was approved by the regional ethics review board (region Arnhem-Nijmegen) under protocol number CMO2015-1813. The study (EudraCT 2015-000917-31) is registered in Dutch trial register no. NL52649.091.15. Written informed consent was obtained from all participants included in this study. All procedures performed in this study were in accordance with the ethical standards of the institutional and national research committee and with the 1964 Helsinki declaration.

**Fig. 1:**
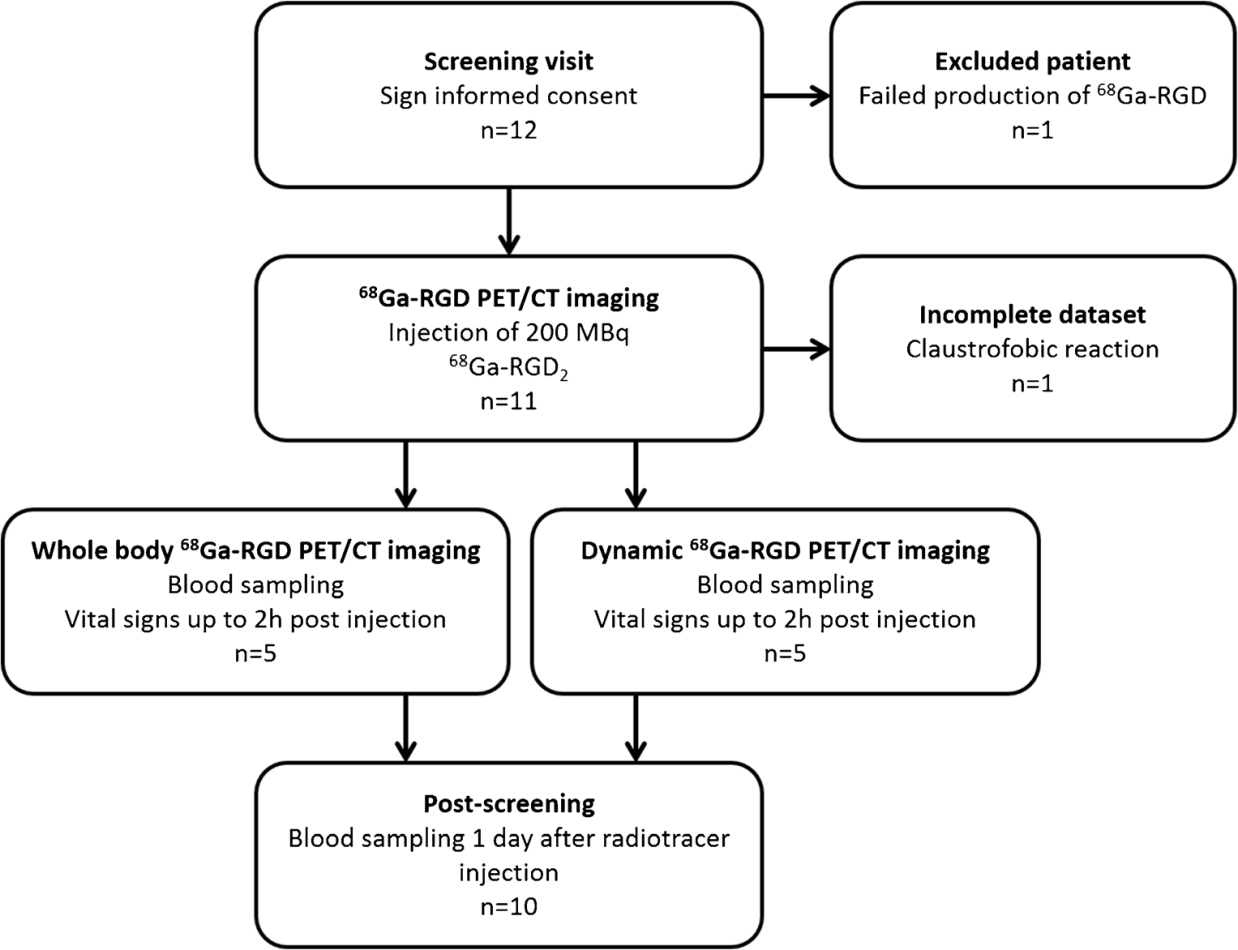
Flowchart of the characteristics of the study design

Twelve patients with proven squamous cell carcinoma of the oral cavity were originally included, of which 10 patients completed the study protocol. All patients were scheduled for surgical resection. Patients were eligible when diagnosed with a tumour diameter of at least 1.5 cm, classified according to radiologic criteria obtained from either MRI or CT within 4 weeks prior to the screening visit. Patients with contra-indications for PET/CT, and/or an impaired renal or liver function (estimated glomerular filtration rate by the modification of diet in renal disease formula (eGFR (MDRD)) ≤ 60 ml/min, aspartate aminotransferase and alanine aminotransferase levels ≥ 3x upper limit of normal, or total bilirubin ≥ 2x upper limit of normal), or when diagnosed with other serious illnesses, were not eligible. ^68^Ga-RGD PET/CT was scheduled 2–7 days prior to the planned surgical intervention. A study flowchart of the design of the study is provided in Fig. 1.

### ^68^Ga-RGD synthesis

DOTA-E-[c(RGDfK)]_2_ was synthesised by coupling E-[c(RGDfK)]_2_ (Peptides International, Louisville, KY, USA) and DOTA(tris)Bu (1,4,7,10-Tetraazacyclododecane-1,4,7,10-tetraacetic acid) (Macrocyclics, Dallas, TX, USA), as described previously [17–19]. Synthesis complied with good manufacturing practice (GMP) regulations for human use. Radiolabelling of DOTA-E-[c(RGDfK)]_2_ was performed on a Scintomics GRP module (Scintomics GmbH, Fürstenfeldbruck, Germany) connected to one or two GMP-grade ^68^Ge/^68^Ga generators (IGG101 GalliaPharm, Eckert and Ziegler, Berlin, Germany). A Scintomics reagent and hardware kit for synthesis of ^68^Ga-peptides using cationic purification (SC-01, ABX, Radeberg, Germany) was used for elution of the ^68^Ge/^68^Ga generator, radiolabelling and purification of [^68^Ga]Ga-DOTA-E-[c(RGDfK)]_2_ (^68^Ga-RGD). After radiosynthesis, quality control of ^68^Ga-RGD was performed according to the European Pharmacopoeia (Ph Eur) standards including verification of radiochemical purity and colloid content using reverse-phase high-performance liquid chromatography and instant thin-layer chromatography, pH determination using a pH indicator strip, assessment of endotoxin content using Endosafe equipment (Charles River, Leiden, The Netherlands) and testing of sterility. All injected preparations met the quality control criteria and had a radiochemical purity > 99%.

### ^68^Ga-RGD PET/CT imaging

Included patients had no restrictions in food or medication intake. ^68^Ga-RGD was administered intravenously using a slow bolus injection of 8 ml over 40 s (200 ± 29 MBq; range 119–218 MBq, 42 ± 8 μg; range 34–58 μg). The net injected dose was calculated by measuring the syringe before and after ^68^Ga-RGD administration using a dedicated dose calibrator (VDS-405, Comecer, Joure, The Netherlands). Patients were monitored for (severe) adverse events according to the Common Terminology Criteria for Adverse Events, version 4.03. Venous blood samples at 1, 2, 5, 10, 30 and 90 min after injection were taken for pharmacokinetic and safety analysis. The concentration of ^68^Ga-RGD in the blood samples (Bq/ml) was calculated by gamma counter (Wizard Detector Gamma Counter, PerkinElmer Inc., Waltham, MA, USA) while simultaneously measuring 1% standards. Safety analysis included a complete blood count test and assessment of kidney and liver function by measuring the eGFR (MDRD), total bilirubin and aspartate aminotransferase and alanine aminotransferase levels.

Five patients underwent three static whole-body PET/CT at 30, 60 and 90 min post injection on a Biograph 40 mCT scanner (Siemens Medical Solutions, Knoxville TN, USA) equipped with a dual-slice CT for 4 min of PET data per bed position, with bed positions from the base of the skull to trochanter major. For each PET/CT image, two image reconstructions were used, with a TrueX algorithm with point spread function (PSF) and time of flight (TOF) measurements, using three iterations, 21 subsets, matrix size 256 × 256 (pixel spacing of 3.2 mm) or 200 × 200 (pixel spacing of 4.07 mm), full width at half maximum (FWHM) of 5 mm. Post-processing was performed using a 3D Gaussian filter kernel, 7.5 or 3 mm. The other five patients underwent 60 min dynamic ^68^Ga-RGD PET/CT starting at start of injection, of the head and neck region (15 cm FOV), to obtain time–activity curves in malignant lesions. These images were reconstructed in 33 frames (1 × 40 s, 10 × 5 s, 3 × 10 s, 2 × 15 s, 5 × 30 s, 5 × 120 s, 5 × 300 s and 2 × 600 s). For all patients, a low-dose CT scan was acquired for attenuation correction and anatomical reference, with an automatically modulated X-ray tube voltage and current (100 kV, 30 mA) using Care kV and Care Dose4D (Siemens Healthcare), 512 × 512 voxels; 0.98 pixel spacing; 3 mm slice thickness; 1 pitch. The CT images for anatomical reference were reconstructed with a B31f convolution kernel, slice thickness 5 mm.

### Tissue and staining

Representative material from tumour and the lymph nodes from the resection specimen of all patients was selected by the pathologist (ACHEG). Four-μm sections, derived from formalin-fixed, paraffin-embedded tissue, were used for staining. Sections were mounted on Superfrost slides (New Silane III, Mutopure Chemicals Co. Ltd., Tokyo, Japan) and subsequently stained for integrin α_v_β_3_ (anti-α_v_β_3_ integrin monoclonal antibody; AB7166, clone BV3, 1:50, Abcam, Cambridge, UK), and for Ki-67 and CD34 using a double staining protocol. A detailed description of the staining protocols can be found in Online Resource 1.

### Image analysis

#### PET data

All ^68^Ga-RGD PET/CT scans were evaluated by two experienced nuclear medicine physicians (WJGO, EHJGA) on a dedicated workstation (Oasis software, Segami Corporation, Columbia, MD, USA) with a PET/CT viewer for automatic image registration. A volume-of-interest (VOI) around the tumour mass was manually drawn to obtain a maximum standardised uptake value (SUV_max_) of the tumour area—drawn on the static PET/CT image acquired 60 min post injection—and to determine the 41% SUV_max_ isocontour and SUV_peak_ (highest mean SUV value of 1 cm^3^) within this tumour volume [20]. The same analysis was applied on the dynamic imaging series, in which a reconstructed frame of 4 min (56–60 min p.i.) was extracted from the dynamic imaging series to obtain a static image for tumour volume delineation. The dynamic imaging series were also used to obtain time–activity curves using the PMOD Medical Imaging Program version 3.15 (PMOD technologies LLC, Zürich, Switzerland). Curves were estimated for both tumour lesions and background tissue. Tumour delineation was based on the evaluation in Oasis (Oasis software, Segami Corporation, Columbia, MD, USA), drawn on the last frame of the PET series, and subsequently copied to the other frames. For time–activity curves of the muscle, a VOI of 1.5 ml was defined in dorsolateral musculature (contralateral to the tumour) using the CT images and subsequently copied to the dynamic dataset. For the time–activity curves of the tracer distribution in the parotid gland and blood pool, VOIs were defined on the CT images within the contralateral parotid gland, carotid artery and jugular vein and subsequently projected on the dynamic PET dataset.

#### Immunohistological analysis

Histological evaluation (HE) and α_v_β_3_ integrin expression pattern were qualitative analysed using light microscopy (Leica DMC2900, Leica Microsystems, Heerbrugg, Switzerland). Besides, an Axio Scope.A1 microscope (Zeiss, Carl Zeiss Bv, The Netherlands) directed by iVision software was used to scan for three signals: DAPI (all nuclei), Alexa-568 (Ki-67) and Alexa-647 (CD34) by means of a 10× objective using standardised shutter times for each signal (5, 500 and 250 ms, respectively). After scanning, grey-scale images of all three recorded signals were used for analysis. Thresholds for segmentation of the fluorescent signals were interactively set above the background staining for each individual marker and adjusted for each sample in order to optimise the signal to background ratio using Fiji (ImageJ, Wayne Rasband, National Institute of Mental Health, National Institutes of Health). The resulting binary images were used to calculate the fraction of Ki-67 relative to the total tumour area (Ki-67 labelling index), and the microvessel density (MVD) as number of vascular structures per mm^2^ using the CD34 staining. To minimise bias of nonspecific staining, only positive signals exceeding 5 pixels (vessels) and 2 pixels (nuclei) were included.

### Statistical analysis

Numerical data are presented as mean ± standard deviation (SD). Patient-based, lesion-based and tissue-based analyses were performed. The biological half-life of ^68^Ga-RGD was calculated using bi-exponential regression analysis in MATLAB version R2014b (MathWorks, Natick, MA, USA). The analyses of optimal imaging time point and uptake values were analysed using SPSS Statistics, version 22.0.0.1 (IBM), with a one-way ANOVA followed by a Bonferroni post hoc test to adjust for multiple comparisons. The correlation between ^68^Ga-RGD uptake, Ki-67 labelling index and MVD was evaluated by linear regression analysis using Graphpad Prism (version 5.03 GraphPad Software), and the Pearson’s correlation coefficient was calculated. In all cases, the level of significance was set to *P* < 0.05.

## Results

### Patient demographics

Ten patients with an OSCC completed all study procedures. Patient characteristics, clinical and pathological tumour classification (TNM classification, 8th edition of the American Joint Committee on Cancer), histology and basic imaging findings of these patients are summarised in Table 1. Five patients underwent whole body ^68^Ga-RGD PET/CT at 30, 60 and 90 min post injection. Four patients underwent 60-min dynamic ^68^Ga-RGD PET/CT, and one patient for 30 min. One patient did not receive ^68^Ga-RGD injection, due to a failed production. One patient experienced severe claustrophobia during scanning, which was considered an adverse event unrelated to ^68^Ga-RGD injection. ^68^Ga-RGD PET/CT was discontinued; no available dataset for analysis was available.

**Table. 1:** Patient characteristics within this study cohort

No.	Age	Gender	Clinical TNM-stage	Imaging protocol	SUV_max_ tumour	Pathological TNM-stage	Tumour volume on PET (ml)	Tumour characterisation (with negative growth characteristics)
1	69	M	cT4aN0M0	Whole body static	6.5	pT4aN2bM0	13	Moderately differentiated SCC (discohesive growth pattern and perineural growth)
2	74	M	cT4aN0M0	Whole body static	7	pT4aN1M0	20	Moderately differentiated SCC
3	71	F	cT3N0M0	Whole body static	5.5	pT4aN0M0	9	Well-differentiated SCC
4	65	M	cT4aN0M0	Whole body static	4.7	pT1N0M0	8	Moderately differentiated SCC (discohesive growth pattern)
5	63	M	cT3N0M0	Dynamic	4	pT2N0M0	8	Moderately differentiated SCC
6	80	F	cT4aN1M0	Dynamic*	8.4	pT4aN3bM0	25	Moderately differentiated SCC (discohesive growth pattern)
7	55	M	cT3N1M0	Dynamic	12.7	pT3N1M0	7	Moderately differentiated SCC (perineural growth)
8	37	F	cT3N0M0	Dynamic	9.7	pT2N3bM0	19	Moderately differentiated SCC
9	70	M	cT3N0M0	Dynamic	6.8	pT2N1M0	20	Moderately differentiated SCC
10	63	M	cT3N0M0	Dynamic	9.4	pT4aN2bM0	20	Moderately differentiated SCC (discohesive growth pattern and perineural growth)

*Patient that only underwent 30-min dynamic scanning

### Safety analysis

All patients were monitored for 2 hours after radiotracer injection and no changes in vital signs nor any (serious) adverse events were observed (data on file). Furthermore, all laboratory values were within normal ranges (data on file).

### Whole blood and plasma concentrations of ^68^Ga-RGD

^68^Ga-RGD rapidly cleared from the blood. The mean levels in whole blood were 27.5 ± 19 kBq/ml, 9.8 ± 6.9 kBq/ml, 6.5 ± 5.0 kBq/ml and 4.7 ± 3.5 kBq/ml, at 5, 30, 60 and 90 min post injection, respectively. The mean concentration of ^68^Ga-RGD in whole blood differed from the mean concentration of ^68^Ga-RGD in plasma (Online Resource 2) indicating that the ^68^Ga-RGD is not taken up by the blood cells. When fitting a bi-exponential curve to the PET/CT data (using all time frames), a distribution half-life (t_1/2α_) of 0.14 ± 0.07 min and 0.70 ± 0.53 min, and an elimination half-life phase (t_1/2β_) of 17.7 ± 8.19 min and 18.6 ± 7.42 min, for arterial blood and venous blood, respectively, were found. No significant difference in the elimination half-life was found between calculation based on blood samples’ measurements (23.0 ± 2.3 min) or based on data derived from PET/CT (t(3) = 0.664, *P* = 0.55).

### Quantitative analysis of ^68^Ga-RGD in tumour and biodistribution in normal tissue

**Fig. 2:**
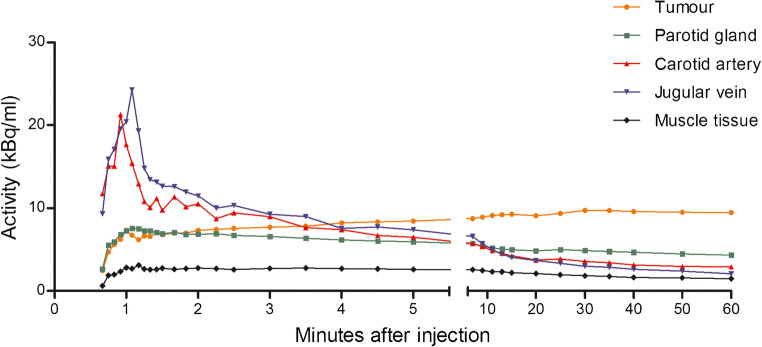
Mean time–activity curves of ^68^Ga-RGD over 60 min after injection in tumour, blood pool, parotid tissue and muscle tissue of patients 6–10. Values are expressed as mean of five patients until 30 min and mean of four patients after 30 min, since one patient prematurely ended dynamic scanning (at 30 min). Data are presented as cumulative data at the end of each frame

The mean SUV_max_ in tumour tissue at 30, 60 and 90 min post injection was 5.7 ± 0.8, 5.5 ± 1.2 and 4.8 ± 1.2 (*n* = 5). These uptake differences between the three imaging time points were not statistically significant (F(2,12) = 0.889, *P* = 0.44). All dynamic PET images showed a slight increase in tracer uptake in tumour tissue during 10 min, followed by a plateau phase up to 60 min post injection. In one patient, the tumour tissue demonstrated a slower increase in tracer accumulation and reached a plateau phase 15 min after injection (Online Resource 3). This tumour showed a distinct morphology on MRI imaging (patient 6) as compared with the other patients, with a severe inflammatory process surrounding the tumour tissue. Muscle tissue activity in the dorsolateral muscles and background parotid gland activity showed no marked changes in radiotracer uptake over time (Fig. 2 and Online Resource 4). Tumour-muscle tissue ratios were higher than 4.0 for every time point from 10 min after injection and increased up to more than 6.0 at the last time point (mean ratio 6.4 ± 0.7). The kidney, liver and bladder showed the highest accumulation of ^68^Ga-RGD in normal tissue and organs (Online Resource 5).

### Immunohistological analysis of α_v_β_3_ integrin expression

**Fig. 3:**
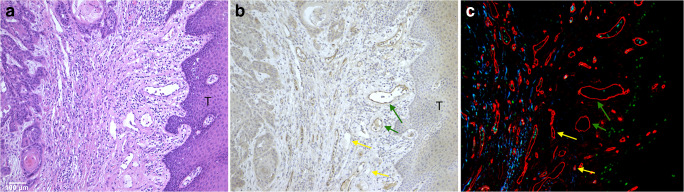
Microscopic images of consecutive tumour sections (× 20objective) stained with haematoxylin and eosin (**a**), anti-α_v_β_3_ integrin (**b**) and double stained for CD34 (red), Ki-67 (green), and DAPI (blue) (**c**). A heterogeneous expression pattern of α_v_β_3_ integrin expressing blood vessels at the tumour invasive front (T) is observed, with both positive en negative stained endothelial cells (green en yellow arrow, resp.)

Thirty-one cell blocks (primary tumours and lymph node metastasis) of the 10 patients were available. A distinct pattern of staining was observed, with integrin α_v_β_3_ predominantly expressed in endothelial cells of tumour-associated blood vessels. As shown in Fig. 3, a heterogeneous distribution of both α_v_β_3_ integrin positive and negative blood vessels was observed. Most positive vessels were observed at the invasive front, of the tumour, but quantitative analysis of this observation was not feasible. In two patients with a squamous cell carcinoma with many keratinization, a strong staining of the tumour cells was observed, whereas other tumours showed no staining of the tumour cells. Furthermore, integrin α_v_β_3_ immunohistochemical staining was to a lesser extent observed in the cytoplasm of tumour and soft tissue. Red blood cells showed nonspecific staining in some samples. The MVD of the primary tumour samples ranged from 155 to 352 mm^−2^ (mean 246 mm^−2^) and the Ki-67 labelling index from 0.1 to 0.9 (mean 0.5). There was no correlation between MVD or Ki-67 labelling index and SUV_41%_ values (*R* = 0.20; *P* = 0.57 and *R* = 0.16; *P* = 0.67, respectively), possibly due to the limited number of study subjects.

### Characterisation of tumour tissue and lymph node metastasis by ^68^Ga-RGD PET

**Fig. 4:**
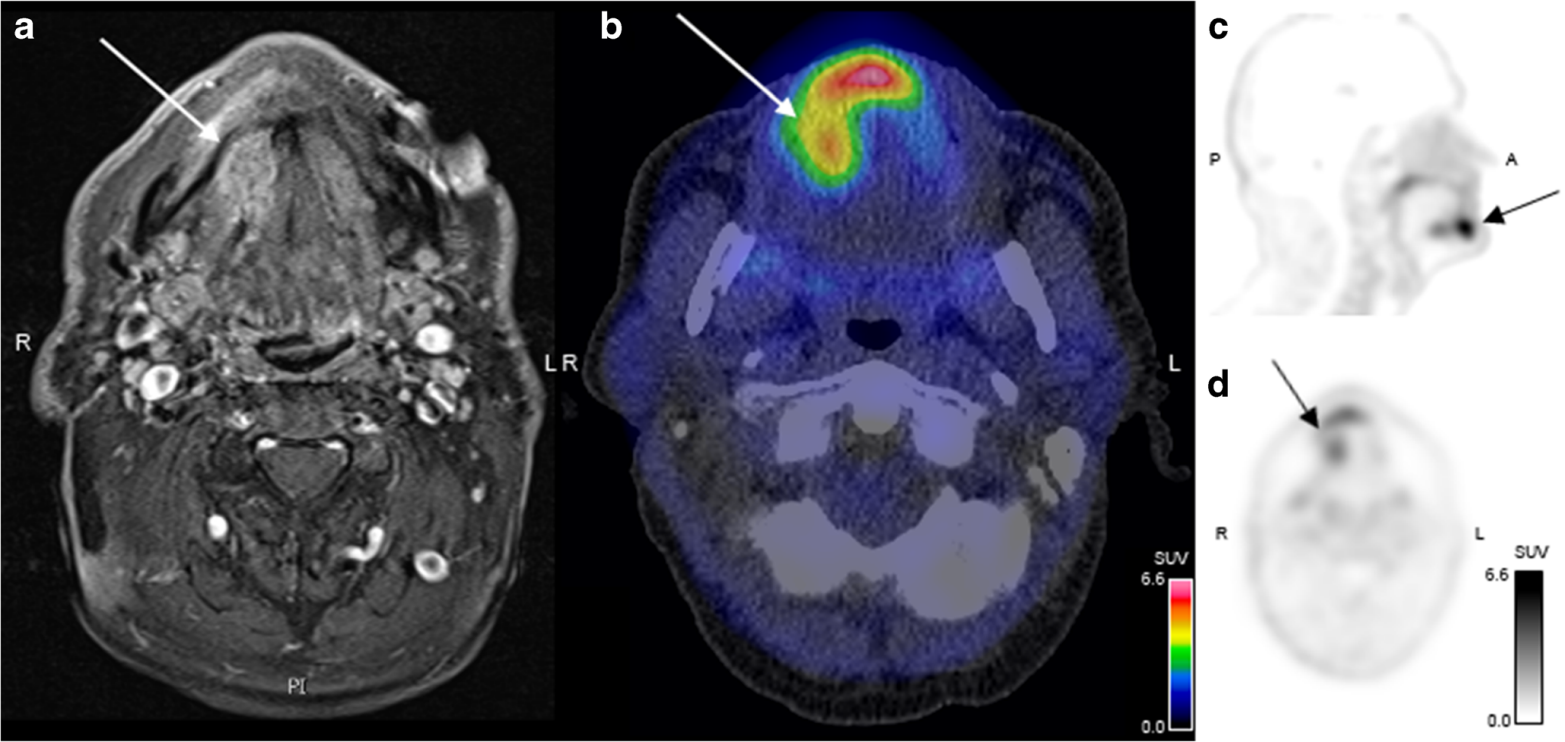
PET/CT after injection of 218 MBq ^68^Ga-RGD in a patient with a moderately differentiated SCC in the right oral cavity. **a** Transverse plane of magnetic resonance image. **b** Fused PET/CT of the oral cavity revealing a tumour lesion with SUV_max_ of 6.5. **c** and **d** Sagittal and axial PET images demonstrating a clearly visualised tumour in the oral cavity

All primary tumours could be identified by ^68^Ga-RGD PET/CT (a representative image is provided in Fig. 4). The mean tumour volume as determined by ^68^Ga-RGD PET/CT was 13.7 ± 5.6 ml. A weak correlation between tumour volume and tumour SUV_max_ was observed (*R* = 0.69, *P* = 0.041), possibly due to partial volume effect.

A total of 321 lymph nodes were dissected of which 13 lymph nodes showed metastasis. We observed radiotracer uptake in six lymph nodes in five patients (mean SUV_max_ 4.4 ± 2.3 (range 1.8–7.6)). The elective neck dissection revealed metastatic disease in 67% (4/6) of the RGD positive lymph nodes. The two lymph nodes with ^68^Ga-RGD accumulation but without confirmed metastatic disease were not suspicious on dynamic contrast-enhanced MRI or CT images. The elective neck dissection revealed an additional nine small lymph node metastases, which remained undetected during preoperative diagnostic imaging, including ^68^Ga-RGD PET/CT. The smallest lymph node metastasis that was detected with ^68^Ga-RGD PET/CT had a diameter of 6 mm.

## Discussion

In this study, we investigated the in vivo biodistribution and kinetics of ^68^Ga-RGD in patients with primary squamous cell carcinomas of the oral cavity prior to resection and correlated these findings with histopathology. The purpose was to evaluate neoangiogenesis in OSCC, rather than the utility of ^68^Ga-RGD PET/CT for staging purposes. The ^68^Ga-RGD PET/CT images revealed variable targeting in all primary tumours. Immunohistological analysis confirmed α_v_β_3_ integrin expression within the HNSCC.

Because data on the in vivo biodistribution and kinetics of the dimeric ^68^Ga-RGD in patients is limited [15, 21] and non-existent for HNSCC, we investigated detailed tissue distribution kinetics in this study. A fast blood clearance was observed using the PET/CT images as image-derived input function, with a venous distribution half-life of 0.14 ± 0.07 min and an elimination half-life phase of 18.6 ± 7.42 min. These results are in line with reported clinical studies on similar radiolabelled RGD peptides in other cancer patients [22], but limited venous blood samples at early time points prevented accurate assessment and validation of the biological clearance rate of ^68^Ga-RGD.

Initial preclinical observations with ^68^Ga-labelled dimeric RGD demonstrated favourable radiochemical characteristics, showing adequate tumour-to-normal tissue ratios and imaging characteristics as compared with monomeric [17, 19] or trimeric RGD compounds [16]. HNSCC lesions were detected with sufficient tumour-to-background ratios and absolute standardised tumour SUV_max_ ranged from 4.7 to 12.7. These findings indicate that higher uptake values can be achieved as compared with, for example, the monomeric ^18^F-Galacto-RGD compound, which showed a mean SUV_max_ of 3.4 ± 1.2 in HNSCC [23]. α_v_β_3_ integrin-mediated uptake in normal tissue was seen in the kidneys, bladder, liver, spleen and intestines [17]. Moreover, uptake in the salivary glands, choroid plexus and thyroid tissue was observed and comparable with similar studies on other radiolabelled RGD peptides [14, 22, 24]. In general, our biodistribution data is in line with the biodistribution data in healthy subjects, reported by Lopez et al. [21]

The large variation in the observed SUV uptake values is most likely the reflection of differences in α_v_β_3_ integrin expression patterns. On the other hand, it may reflect a combination of radiotracer binding to neovasculature or tumour cells. In previous studies, it was reported that HNSCCs express α_v_β_3_ integrins solely on the neovasculature of tumours [10, 23], while other solid tumours, e.g. renal cell carcinomas, also demonstrated α_v_β_3_ integrin expression on the tumour cells themselves [25, 26]. The current study identified additional staining of tumour cells in two tumours with highly keratinizing OSCC phenotype which did not result in higher SUV values. Although this could be nonspecific, this might also indicate that histological subtypes of cancer have distinct expression patterns of α_v_β_3_ integrin. Similar findings were also reported by Beer et al. [23] Furthermore, the PET/CT findings may reflect ^68^Ga-RGD binding to other subtypes of integrins (e.g. α_5_β_1_ or α_v_β_5_), which have showed its overexpression in several types of HNSCC [27]. RGD-based peptides may bind other integrins, although with a lower affinity, unlikely to have a significant effect on the quantification of binding to α_v_β_3_ integrins [11, 28]. Conversely, neovasculature is not only a hallmark of cancer, it is also essential in the process of (chronic) inflammatory processes [29]. These α_v_β_3_ integrin expression patterns could potentially interfere with the interpretation of the RGD PET/CT findings of the tumour neovasculature in and surrounding the tumour microenvironment. Further investigation in the expression pattern of α_v_β_3_ integrin and whether RGD PET/CT is a valid measure of endothelial cell activation in tumours might be relevant, especially in those molecular imaging studies aiming to determine changes in angiogenesis during treatment using RGD PET/CT.

While all primary tumours could be identified on ^68^Ga-RGD PET/CT, not all lymph node metastases were identified. Small lesions may be difficult to detect due to the limited spatial resolution of PET/CT. On the other hand, the detection rate may be limited due to low α_v_β_3_ integrin expression patterns in metastatic lesions as compared with primary tumour tissue. RGD uptake tends to be higher in those lymph nodes with large metastases than those without confirmed metastatic disease or smaller lymph node metastases (< 5 mm). Although to lesser extent than in tumours, α_v_β_3_ integrins are also expressed during inflammation, e.g. on endothelial cells and activated macrophages, which might in part explain uptake in reactive lymph nodes that drain the primary tumour area.

A limitation of this study is the small number of patients with OSCC included, which does not fully reflect the heterogeneity of HNSCC, with its many different biological characteristics. Microenvironmental differences and their effect on ^68^Ga-RGD PET/CT need to be elucidated in further studies and may allow more detailed analysis of the variability in SUV values and integrin α_v_β_3_ expression pattern across the patients. The ^68^Ga-RGD PET/CT findings may be of interest to differentiate between HNSCC subtypes and may allow stratification of those patients who will most likely respond to conventional treatment or treatment with angiogenic inhibitors [27]. Previously, a preclinical and clinical pilot study have demonstrated the feasibility of treatment response monitoring in HNSCC xenografts and patients using RGD PET/CT [30, 31], but it is required to study this in prospective clinical trials with more patients.

In conclusion, the results of this study support the feasibility of ^68^Ga-RGD PET/CT as a molecular imaging technique of α_v_β_3_ integrin expression in OSCC. It is a potential candidate to evaluate tumour angiogenesis during treatment and may also provide more insight in the vasculature of the distinct tumour microenvironments of HNSCC.

## Electronic supplementary material

ESM 1 (DOCX 1044 kb)
