# Supplementary material for: Imaging angiogenesis in patients with head and neck squamous cell carcinomas by [68Ga]Ga-DOTA-E-[c(RGDfK)]2 PET/CT
Source: Eur J Nucl Med Mol Imaging. 2020 Mar 20;47(11):2647–55. doi: 10.1007/s00259-020-04766-2 (PMC7515959; doi:10.1007/s00259-020-04766-2)
Supplement: Supplementary file 1 — (DOCX 1044 kb) [file 259_2020_4766_MOESM1_ESM.docx]

EJNMMI Online Resource 1

Title: Imaging angiogenesis in patients with head and neck squamous cell carcinomas by [^68^Ga]Ga-DOTA-E-[c(RGDfK)]_2_ PET/CT

Authors: D. Lobeek^1^, M. Rijpkema^1^, S.Y.A. Terry^2^, J.D.M. Molkenboer-Kuenen^1^, L. Joosten^1^, E.A.J. van Genugten^1,3^, A.C.H. van Engen-van Grunsven^4^, J.H.A.M. Kaanders^5^, S.A.H. Pegge^1^, O.C. Boerman^1^, W.L.J. Weijs^6^, M.A.W. Merkx^6^, C.M.L. van Herpen^7^, R.P. Takes^8^, E.H.J.G. Aarntzen^1^, W.J.G. Oyen^1,9,10^

Affiliations:

^1^Department of Radiology and Nuclear Medicine, Radboud University Medical Center Nijmegen, Nijmegen, The Netherlands

^2^Department of Imaging Chemistry and Biology, King’s College London, London, United Kingdom

^3^MIRA Institute for Biomedical Technology and Technical Medicine, University of Twente, Enschede, The Netherlands

^4^Department of Pathology, Radboud University Medical Center Nijmegen, Nijmegen, The Netherlands

^5^Department of Radiotherapy, Radboud University Medical Center Nijmegen, Nijmegen, The Netherlands

^6^Department of Oral and Maxillofacial Surgery, Radboud University Medical Center, Nijmegen

^7^Department of Medical Oncology, Radboud University Medical Center Nijmegen, Nijmegen, The Netherlands

^8^Department of Otolaryngology/Head and Neck Surgery, Radboud University Medical Center, Nijmegen

^9^Department of Biomedical Sciences, Humanitas University, Milan, Italy

^10^Department of Radiology and Nuclear Medicine, Rijnstate Hospital, Arnhem, The Netherlands

Correspondence to: D. Lobeek (daphne.lobeek@radboudumc.nl)

Addition information on Immunohistochemical staining

All slides were deparaffinated and rehydrated (graded ethanol: 100-70-50% and de-ionised water).

*α_v_β_3_ staining on paraffin sections*

For the α_v_β_3_ integrin staining, antigen retrieval was performed in 10 mM TRIS-EDTA pH9+0.05% Tween20 for 10 min at 95°C. The sections were blocked with 3% peroxidase in PBS and then blocked using Avidin/Biotin blocking kit (SP-2001, Vector). After blocking, all sections were pre-incubated with 20% Normal Goat Serum (NGS) for 30 min at RT and the primary mouse-anti-human α_v_β_3_ was applied for overnight incubation at 4°C. The next day, after incubation with secondary antibody biotinylated goat-anti-mouse IgG (1:100; BA9200, Vector labs), all sections were incubated with pre-incubated Biotin/Streptavidin complex (ABC-kit, Vectastain, USA) for 30 min at RT (dark). All sections were subsequently washed three times with 50 mM PBS and stained with Bright DAB solution (Immunologic, Duiven, The Netherlands) at RT (dark) for 8 min and washed with tapwater. Finally, all sections were counterstained with haematoxylin solution for 2 seconds, washed in tapwater for 10 min, and mounted with Permount^TM^ mounting medium. Controls consisted of incubations replacing the primary antibodies with phosphate-buffered saline (PBS) + 1% Bovine Serum Albumine (BSA).

*CD34 and Ki-67 double staining protocol on paraffin sections*

A double staining protocol for Ki-67 and CD34 staining was applied. A citrate buffer antigen retrieval was performed for 30 min (Target retrieval solution 10×, pH 6 citrate, Dako Cytomation, 96 °C). After sections were blocked with 20% normal goat serum (NGS), 30 min at room temperature, sections were incubated with mouse-anti-CD34 (1:200, QBend-10 ab8536, Abcam, Cambridge, UK)) for 60 min. Subsequently, sections were blocked with 20% normal donkey serum (NDS) for 30 min and staining was continued by a 60 min staining for Ki-67 (1:50, rabbit-anti-ki67 (RM-9106-S1, ThermoFisher, Waltham, Massachusetts, USA)) at room temperature. Secondary incubation was performed using Alexa-647-goat-anti-mouse (1:200, Alexa Fluor™ 647 IgG2b, ThermoFisher, Waltham, Massachusetts, USA) for CD34 and Alexa-568-donkey-anti-rabbit (1:200, Rabbit IgG (H+L) Highly Cross-Adsorbed Secondary Antibody A10042, ThermoFisher, Waltham, Massachusetts, USA) for Ki-67. The primary antibodies were diluted in PBS + 0.05% Tween 20, the blocking serums were diluted in PBS, and both secondary antibodies were diluted in PBS + 1% BSA. In between stainings, sections were rinsed with PBS. 4′,6-Diamidino-2-phenylindole (DAPI) (32670 BioReagent, Sigma-Aldrich, Saint Louis, Missouri, USA) was used as a counterstain to stain all nuclei. Finally the sections were mounted with Fluoromount (Fluoromount™ Aqueous Mounting, Sigma-Aldrich, Saint Louis, Missouri, USA). Haematoxylin and eosin staining was used for histological evaluation.

EJNMMI Online Resource 2

Title: Imaging angiogenesis in patients with head and neck squamous cell carcinomas by [^68^Ga]Ga-DOTA-E-[c(RGDfK)]_2_ PET/CT

Authors: D. Lobeek^1^, M. Rijpkema^1^, S.Y.A. Terry^2^, J.D.M. Molkenboer-Kuenen^1^, L. Joosten^1^, E.A.J. van Genugten^1,3^, A.C.H. van Engen-van Grunsven^4^, J.H.A.M. Kaanders^5^, S.A.H. Pegge^1^, O.C. Boerman^1^, W.L.J. Weijs^6^, M.A.W. Merkx^6^, C.M.L. van Herpen^7^, R.P. Takes^8^, E.H.J.G. Aarntzen^1^, W.J.G. Oyen^1,9,10^

Affiliations:

^1^Department of Radiology and Nuclear Medicine, Radboud University Medical Center Nijmegen, Nijmegen, The Netherlands

^2^Department of Imaging Chemistry and Biology, King’s College London, London, United Kingdom

^3^MIRA Institute for Biomedical Technology and Technical Medicine, University of Twente, Enschede, The Netherlands

^4^Department of Pathology, Radboud University Medical Center Nijmegen, Nijmegen, The Netherlands

^5^Department of Radiotherapy, Radboud University Medical Center Nijmegen, Nijmegen, The Netherlands

^6^Department of Oral and Maxillofacial Surgery, Radboud University Medical Center, Nijmegen

^7^Department of Medical Oncology, Radboud University Medical Center Nijmegen, Nijmegen, The Netherlands

^8^Department of Otolaryngology/Head and Neck Surgery, Radboud University Medical Center, Nijmegen

^9^Department of Biomedical Sciences, Humanitas University, Milan, Italy

^10^Department of Radiology and Nuclear Medicine, Rijnstate Hospital, Arnhem, The Netherlands

Correspondence to: D. Lobeek (daphne.lobeek@radboudumc.nl)


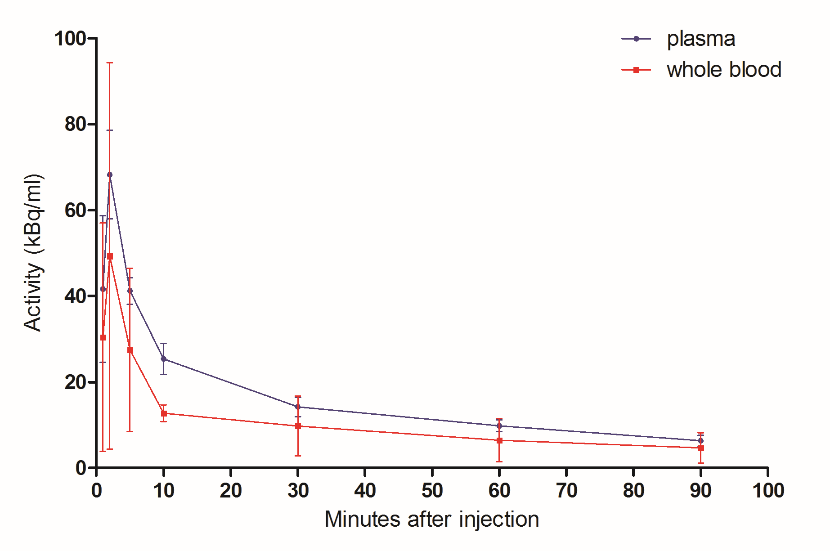


**Online Resource 2** ^68^Ga-RGD tracer clearance for whole blood and blood plasma samples

EJNMMI Online Resource 3

Title: Imaging angiogenesis in patients with head and neck squamous cell carcinomas by [^68^Ga]Ga-DOTA-E-[c(RGDfK)]_2_ PET/CT

Authors: D. Lobeek^1^, M. Rijpkema^1^, S.Y.A. Terry^2^, J.D.M. Molkenboer-Kuenen^1^, L. Joosten^1^, E.A.J. van Genugten^1,3^, A.C.H. van Engen-van Grunsven^4^, J.H.A.M. Kaanders^5^, S.A.H. Pegge^1^, O.C. Boerman^1^, W.L.J. Weijs^6^, M.A.W. Merkx^6^, C.M.L. van Herpen^7^, R.P. Takes^8^, E.H.J.G. Aarntzen^1^, W.J.G. Oyen^1,9,10^

Affiliations:

^1^Department of Radiology and Nuclear Medicine, Radboud University Medical Center Nijmegen, Nijmegen, The Netherlands

^2^Department of Imaging Chemistry and Biology, King’s College London, London, United Kingdom

^3^MIRA Institute for Biomedical Technology and Technical Medicine, University of Twente, Enschede, The Netherlands

^4^Department of Pathology, Radboud University Medical Center Nijmegen, Nijmegen, The Netherlands

^5^Department of Radiotherapy, Radboud University Medical Center Nijmegen, Nijmegen, The Netherlands

^6^Department of Oral and Maxillofacial Surgery, Radboud University Medical Center, Nijmegen

^7^Department of Medical Oncology, Radboud University Medical Center Nijmegen, Nijmegen, The Netherlands

^8^Department of Otolaryngology/Head and Neck Surgery, Radboud University Medical Center, Nijmegen

^9^Department of Biomedical Sciences, Humanitas University, Milan, Italy

^10^Department of Radiology and Nuclear Medicine, Rijnstate Hospital, Arnhem, The Netherlands

Correspondence to: D. Lobeek (daphne.lobeek@radboudumc.nl)

**
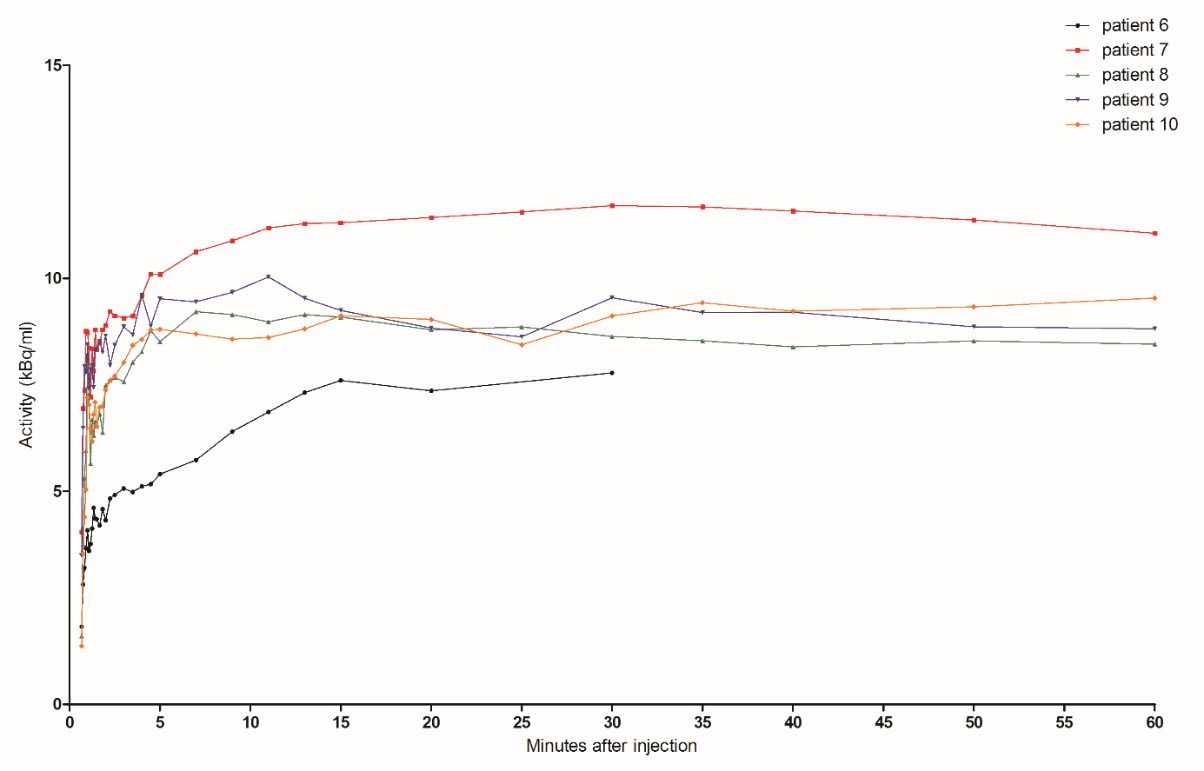
**

**Online Resource 3** Time-activity curves of ^68^Ga-RGD over 60 min after injection in tumour tissue of patients 7-10 and over 30 min after injection of patient 6. Data are presented as cumulative data at the end of each frame

EJNMMI Online Resource 4

Title: Imaging angiogenesis in patients with head and neck squamous cell carcinomas by [^68^Ga]Ga-DOTA-E-[c(RGDfK)]_2_ PET/CT

Authors: D. Lobeek^1^, M. Rijpkema^1^, S.Y.A. Terry^2^, J.D.M. Molkenboer-Kuenen^1^, L. Joosten^1^, E.A.J. van Genugten^1,3^, A.C.H. van Engen-van Grunsven^4^, J.H.A.M. Kaanders^5^, S.A.H. Pegge^1^, O.C. Boerman^1^, W.L.J. Weijs^6^, M.A.W. Merkx^6^, C.M.L. van Herpen^7^, R.P. Takes^8^, E.H.J.G. Aarntzen^1^, W.J.G. Oyen^1,9,10^

Affiliations:

^1^Department of Radiology and Nuclear Medicine, Radboud University Medical Center Nijmegen, Nijmegen, The Netherlands

^2^Department of Imaging Chemistry and Biology, King’s College London, London, United Kingdom

^3^MIRA Institute for Biomedical Technology and Technical Medicine, University of Twente, Enschede, The Netherlands

^4^Department of Pathology, Radboud University Medical Center Nijmegen, Nijmegen, The Netherlands

^5^Department of Radiotherapy, Radboud University Medical Center Nijmegen, Nijmegen, The Netherlands

^6^Department of Oral and Maxillofacial Surgery, Radboud University Medical Center, Nijmegen

^7^Department of Medical Oncology, Radboud University Medical Center Nijmegen, Nijmegen, The Netherlands

^8^Department of Otolaryngology/Head and Neck Surgery, Radboud University Medical Center, Nijmegen

^9^Department of Biomedical Sciences, Humanitas University, Milan, Italy

^10^Department of Radiology and Nuclear Medicine, Rijnstate Hospital, Arnhem, The Netherlands

Correspondence to: D. Lobeek ([daphne.lobeek@radboudumc.nl](mailto:daphne.lobeek@radboudumc.nl))

**
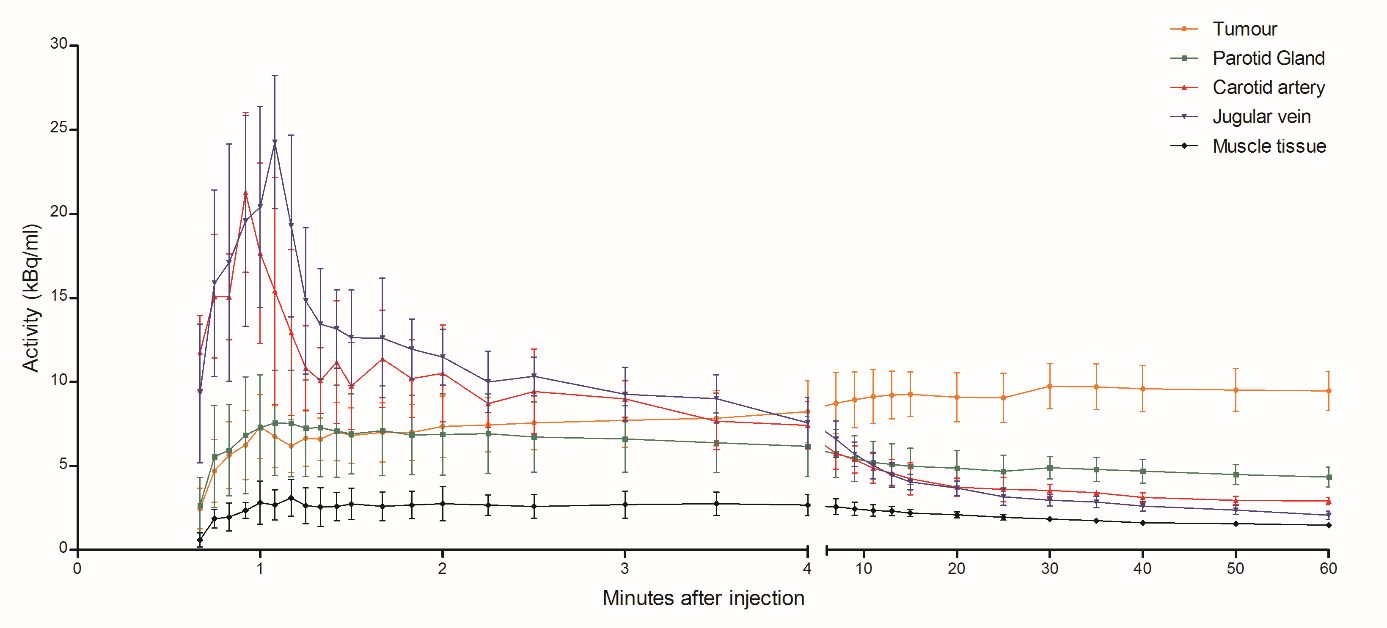
**

**Online Resource 4** Mean time-activity curves including standard deviation of ^68^Ga-RGD in tumour, blood pool, parotid tissue, and muscle tissue. Values are expressed as mean of five patients until 30 min, since one patient prematurely ended dynamic scanning (at 30 min). Data are presented as cumulative data at the end of each frame

EJNMMI Online Resource 5

Title: Imaging angiogenesis in patients with head and neck squamous cell carcinomas by [^68^Ga]Ga-DOTA-E-[c(RGDfK)]_2_ PET/CT

Authors: D. Lobeek^1^, M. Rijpkema^1^, S.Y.A. Terry^2^, J.D.M. Molkenboer-Kuenen^1^, L. Joosten^1^, E.A.J. van Genugten^1,3^, A.C.H. van Engen-van Grunsven^4^, J.H.A.M. Kaanders^5^, S.A.H. Pegge^1^, O.C. Boerman^1^, W.L.J. Weijs^6^, M.A.W. Merkx^6^, C.M.L. van Herpen^7^, R.P. Takes^8^, E.H.J.G. Aarntzen^1^, W.J.G. Oyen^1,9,10^

Affiliations:

^1^Department of Radiology and Nuclear Medicine, Radboud University Medical Center Nijmegen, Nijmegen, The Netherlands

^2^Department of Imaging Chemistry and Biology, King’s College London, London, United Kingdom

^3^MIRA Institute for Biomedical Technology and Technical Medicine, University of Twente, Enschede, The Netherlands

^4^Department of Pathology, Radboud University Medical Center Nijmegen, Nijmegen, The Netherlands

^5^Department of Radiotherapy, Radboud University Medical Center Nijmegen, Nijmegen, The Netherlands

^6^Department of Oral and Maxillofacial Surgery, Radboud University Medical Center, Nijmegen

^7^Department of Medical Oncology, Radboud University Medical Center Nijmegen, Nijmegen, The Netherlands

^8^Department of Otolaryngology/Head and Neck Surgery, Radboud University Medical Center, Nijmegen

^9^Department of Biomedical Sciences, Humanitas University, Milan, Italy

^10^Department of Radiology and Nuclear Medicine, Rijnstate Hospital, Arnhem, The Netherlands

Correspondence to: D. Lobeek (daphne.lobeek@radboudumc.nl)


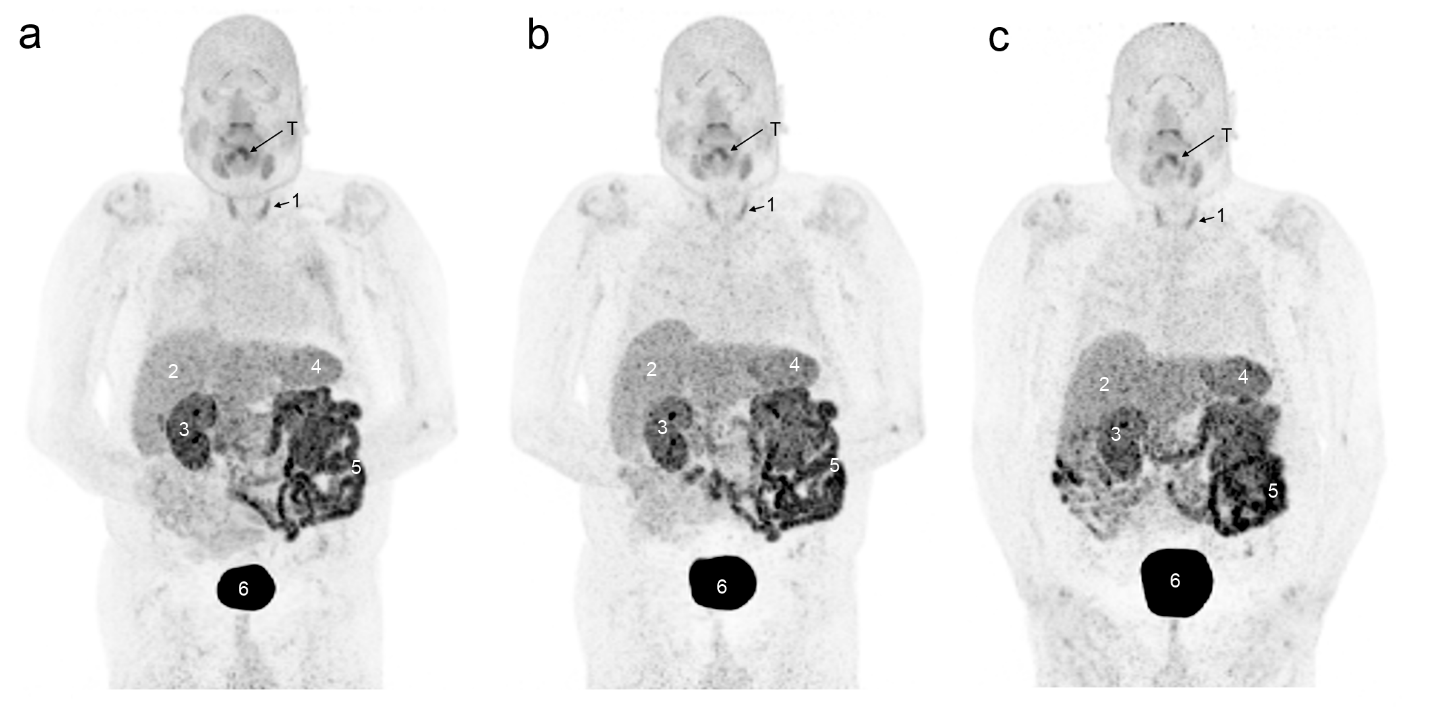


**Online Resource 5** (A-C) Biodistribution of ^68^Ga-RGD in a patient with a pT2N1M0 moderately differentiated squamous cell carcinoma in the oral cavity (T) and non-targeted organs thyroid (1), liver (2), kidneys (3), spleen (4), intestines (5), and bladder (6), at 30 (a), 60 (b), and 90 (c) minutes after intravenous tracer injection
